# Dementia in a patient with Thymoma and hypogammaglobulinaemia (Good's syndrome)

**DOI:** 10.1186/1757-1626-1-90

**Published:** 2008-08-13

**Authors:** Nelson Pedro Ferreira de Jesus, Patrícia Margarida Serra Carvalho, Florbela Maria Grilo Dias, Elsa Maria Filipe Gaspar, José Júlio Alves de Moura

**Affiliations:** 1 Hospitais da Universidade Coimbra, Serviço de Medicina II, Av. Bissaya Barreto e Praceta Prof. Mota Pinto, 3000-075, Coimbra, Portugal

## Abstract

Good's syndrome is extremely rare and refers to an acquired B and T cell immunodeficiency in thymoma patients. The authors of this article present a case report of a 75-year-old, caucasian male patient previously subjected to examinations for secondary dementia and recurrent infections, which revealed paraneoplastic syndrome arose from thymoma. He underwent thymectomy, while his immunodeficiency syndrome sustained with frequent opportunistic infections, constantly requiring intravenous immunoglobulin treatment.

## Background

Although thymoma is the most frequent primary neoplasm in the anterior mediastinum in adult patients, in fact is a rare malignant neoplasia.

Good's syndrome is the association of thymoma with immunodeficiency, characterized by hypogammaglobulinaemia, depleted B-cells, diminished T-cells and inversion of the CD4/CD8 ratio [1].

Although there are formal diagnostic criteria for this disorder, it is classified as a distinct entity by the expert committee of the Word Health Organization/International Union of Immunological Societies on primary immunodeficiencies [2].

In patients with thymoma, the incidence of hypogammaglobulinaemia is 6–11% [3,4].

Thymectomy usually favourably affects associated conditions, such as pure red cell aplasia, but does not improve hypogammaglobulinaemia, thus the patient remains dependent on intravenous immunoglobulin and prone to infections.

This report presents a case of a patient admitted to hospital for dementia examinations, whose results establish the diagnosis of type-A medullary thymoma associated with parapneoplasic syndrome.

## Case presentation

We present a case of a 75-year-old caucasian male patient with recurrent emergency visits in 2007, with prolonged febrile illness, recurring infections, anorexia, weight loss (± 18 Kg in 3 months), cough with muco-purulent sputum and intermittent diarrhoea with one year evolution, simultaneously with symptoms of rapidly progressive dementia.

After several observations in emergency room, the patient was finally admitted to the 2^nd ^Department of Medicine of University Hospital of Coimbra in June, 2007, with chronic diarrhoea and secondary dementia.

On physical examination, the patient was dehydrated, malnourished, with temperature of 37.3 degree C and presented accentuated cognitive deficiency (mini-mental score 15).

**Figure 1:**
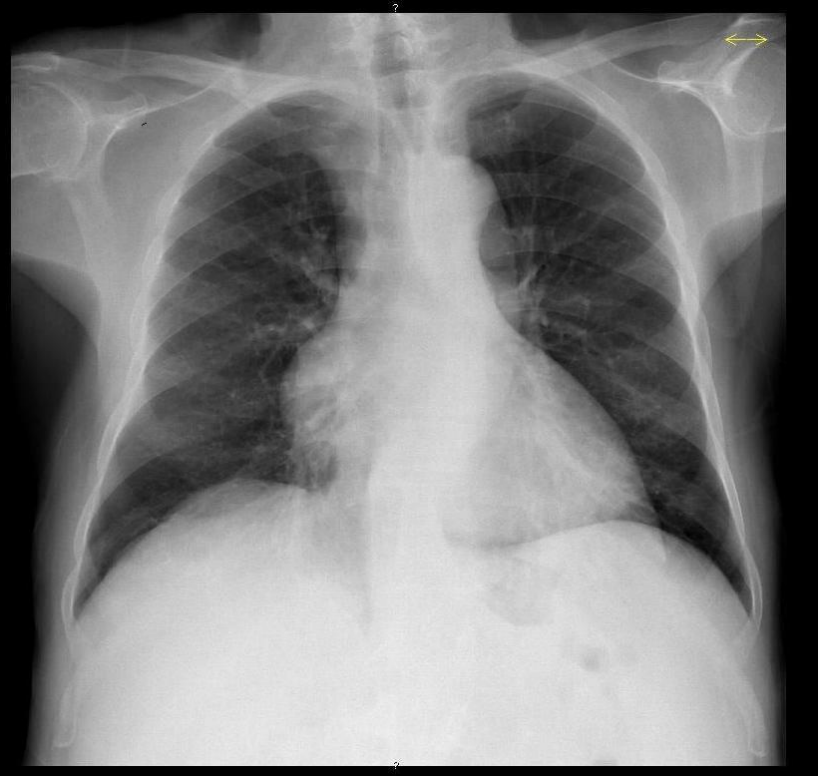
chest X-ray; mediastinal enlargement detected in the initial study of the patient.

**Figure 2:**
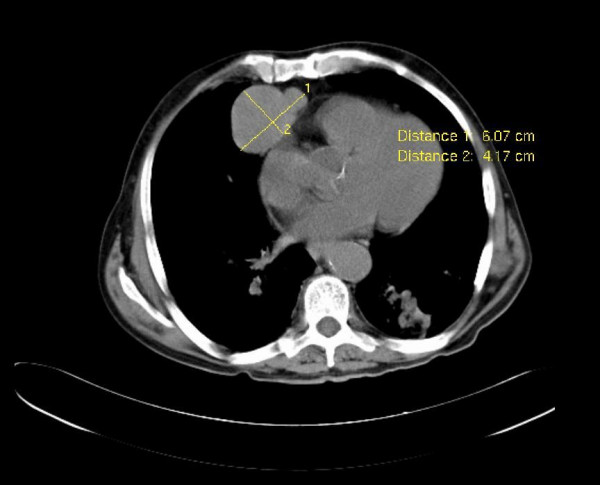
**CT scan; CT scan confirmed mediastinal abnormality, demonstrating a 6-cm mass in the anterior mediastinum**.

Investigations revealed a WBC count of 1,700 cells/L, (differential cell count: 72% neutrophils; lymphocyte study showed 6/cmm); lymphocyte study with 6/mm^3 ^of B lymphocytes, 228/mm^3 ^of CD4+ T cells and CD4/CD8 ratio of 0,63; renal, hepatic and thyroid function, folic acid and B12 vitamin levels were normal. The immunophenotype study of bone marrow revealed cytopenia with multiline dysplasia. Antinuclear antibody and HIV1/2 serologies were negative. Stool culture was negative for ova and parasites. The proteinogram showed hypogammaglobulinaemia with IgG: 0,23 g/L; IgA <0,06 g/L and IgM <0,04 g/L. The cerebral perfusion scintigraphy showed diffuse cerebral hypoperfusion and the brain CT scan revealed generalized cortical-subcortical atrophy. The study of the cerebrospinal fluid (CSF) did not reveal any changes even though oligoclonal bands were detected in the CSF and serum. The electroencephalogram indicated left-sided temporo-occipatal dysfunction with slow focal activity. The chest X-ray showed mediastinal enlargement (figure 1). The thoracic-abdominal-pelvic CT scan revealed a hypodense lobulated nodule in the anterior mediastinum, 66 mm in diameter, with iodinated contrast enhancement (figure 2).

**Figure 3:**
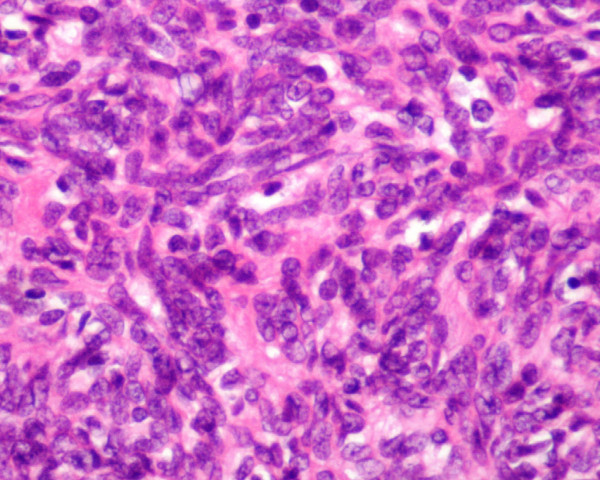
**Histology; Type-A Medullary Thymoma in WHO Classification**.

After the detection of the mediastinal mass and the hypogammaglobulinaemia likely diagnosis of thymoma was made, the patient underwent surgical biopsy and the histology result showed Type-A Medullary Thymoma (figure 3).

The surgical excision of the tumour was done in August, 2007.

After thymectomy, the patient continued having hypogammaglobulinaemia and recurrent infections (respiratory and gastrointestinal), which necessitated further hospitalization. IV Imunoglobulin therapy was started, with slight improvement.

Currently, the patient is entirely dependent and requires recurrent hospitalization, mostly due to infection.

## Discussion

Thymomas are associated with different paraneoplasic syndromes like myasthenia gravis, Lambert-Eaton myasthenia syndrome, pemphigus, subacute sensory neuronopathy, pure red cell aplasia and immunodeficiency [5]. The rapidly progressive dementia in this patient was eventually an unusual paraneoplasic manifestation.

Good's syndrome (thymoma with immunodeficiency) is associated with higher susceptibility to infections, as we could see in this case, even after surgical resection of the tumour, which is explained by the lack of improvement of the hypogammaglobulinaemia after thymectomy. This histological type of thymoma has a good prognosis if isolated, whereas it has a poor outcome if associated to infectious, auto-immune and haematological complications of Good's syndrome.

## Competing interests

The authors declare that they have no competing interests.

## Authors' contributions

All authors provided clinical and scientific orientation in this case report; all authors read and approved the final manuscript.

## Consent

Written informed consent was obtained from the patient's wife (due to patient disability) for publication of this case report and accompanying images. A copy of the written consent is available for review by the Editor-in-Chief of this journal.
